# Chromoblastomycosis: A Case of a Verrucous Plaque from the Tropics

**DOI:** 10.4269/ajtmh.20-0243

**Published:** 2020-08

**Authors:** Paloma M. Carcamo, Alvaro Schwalb, Carlos Seas

**Affiliations:** 1 School of Medicine, Universidad Peruana Cayetano Heredia, Lima, Peru;; 2 Instituto de Medicina Tropical Alexander von Humboldt, Universidad Peruana Cayetano Heredia, Lima, Peru

**Figure 1.**
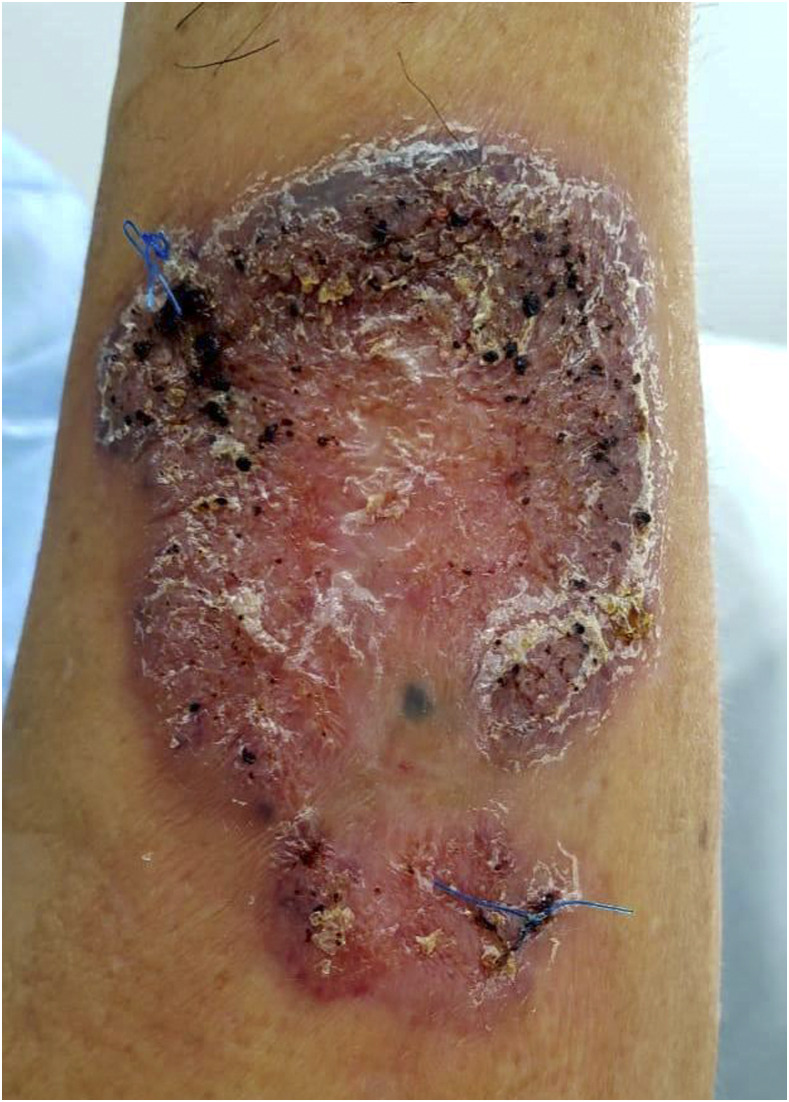
An elevated verrucous plaque with central atrophy and peripheral desquamation, with numerous black dots. This figure appears in color at www.ajtmh.org.

**Figure 2.**
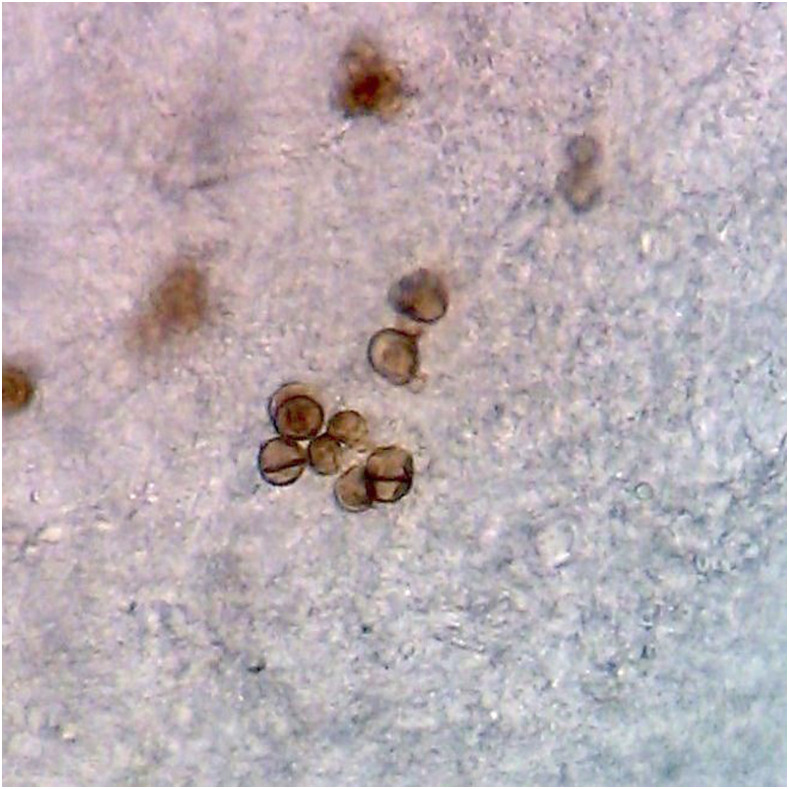
Muriform bodies in microscopic observation of scraping of the skin lesion with 10% KOH. This figure appears in color at www.ajtmh.org.

A 65-year-old man from a rural town in Ucayali, in the Peruvian Amazon jungle, presented to the infectious disease outpatient clinic at Hospital Cayetano Heredia, with a 6-year history of illness characterized by a cutaneous lesion in his left forearm that caused mild pruritus. He had previously seen various medical specialists and received treatment with pentavalent antimonials for suspected cutaneous leishmaniasis and standard regimen for suspected cutaneous tuberculosis, with no improvement. He worked at a farm where he was involved in manual agricultural labor. Physical examination revealed a slightly elevated, dry, verrucous plaque with an atrophic center, peripheral desquamation, and numerous black dots (Figure 1). Direct microscopy of a skin scraping with 10% KOH revealed abundant muriform cells (Figure 2), also known as Medlar bodies, which are pathognomonic for chromoblastomycosis.

Chromoblastomycosis is caused by the inoculation of spores from dematiaceous fungi, primarly from *Fonsecaea* and *Cladophialophora*. It is prevalent in Southern Africa and some parts of Latin America, especially Brazil.^1^ Its prevalence in Peru has not been thoroughly studied, although sporadic cases have been reported in the Amazon region, specifically in Cusco and Ucayali.^2^ Common causes of verrucous plaques in the tropics include cutaneous tuberculosis, leishmaniasis, sporotrichosis, and leprosy, but the black dots in the lesions are a characteristic of chromoblastomycosis and represent the transdermal elimination of debris which provide the best yield for fungal structures.^3^ About 90% of isolated species are identified as *Fonsecaea pedrosoi*.^4^ Treatment involves the prolonged use of oral itraconazole and adjuvant physical methods such as surgical excision, heat or cold therapy, and even laser ablation.^1^ Our patient is currently receiving treatment with oral itraconazole and will be periodically monitored for clinical improvement.
